# Origin of rebound virus in chronically SIV-infected Rhesus monkeys following treatment discontinuation

**DOI:** 10.1038/s41467-020-19254-2

**Published:** 2020-10-27

**Authors:** Po-Ting Liu, Brandon F. Keele, Peter Abbink, Noe B. Mercado, Jinyan Liu, Esther A. Bondzie, Abishek Chandrashekar, Erica N. Borducchi, Joseph Hesselgesser, Michael Mish, Gregory Chin, Elena Bekerman, Romas Geleziunas, Dan H. Barouch

**Affiliations:** 1 Center for Virology and Vaccine Research, Beth Israel Deaconess Medical Center, Harvard Medical School, Boston, MA USA; 2 AIDS and Cancer Virus Program, Frederick National Laboratory for Cancer Research, Frederick, MD USA; 3 Gilead Sciences, Foster City, CA USA; 4 Ragon Institute of Massachusetts General Hospital, Massachusetts Institute of Technology, and Harvard University, Cambridge, MA USA

**Keywords:** RNA sequencing, Retrovirus, Viral reservoirs

## Abstract

Viral rebound following antiretroviral therapy (ART) discontinuation in HIV-1-infected individuals is believed to originate from a small pool of CD4+ T cells harboring replication-competent provirus. However, the origin and nature of the rebound virus has remained unclear. Recent studies have suggested that rebound virus does not originate directly from individual latent proviruses but rather from recombination events involving multiple proviruses. Here we evaluate the origin of rebound virus in 16 ART-suppressed, chronically SIV-infected rhesus monkeys following ART discontinuation. We sequence viral RNA and viral DNA in these animals prior to ART initiation, during ART suppression, and following viral rebound, and we compare rebound viral RNA after ART discontinuation with near full-length viral DNA from peripheral blood and lymph node mononuclear cells (PBMC and LNMC) during ART suppression. Sequences of initial rebound viruses closely match viral DNA sequences in PBMC and LNMC during ART suppression. Recombinant viruses are rare in the initial rebound virus populations but arise quickly within 2–4 weeks after viral rebound. These data suggest that intact proviral DNA in PBMC and LNMC during ART suppression is likely the direct origin of viral rebound in chronically SIV-infected rhesus monkeys following ART discontinuation.

## Abstract (web summary)

The origin and nature of rebound HIV-1 virus following antiretroviral therapy (ART) discontinuation still remains unclear. Here, Liu et al. suggest that intact proviral DNA in peripheral blood and lymph node mononuclear cells during ART suppression likely is the source of viral rebound following ART discontinuation.

## Introduction

ART leads to durable suppression of HIV-1 replication in the majority of individuals, but the persistent viral reservoir in resting memory CD4+ T cells is the key barrier to HIV-1 cure and leads to viral rebound in the vast majority of HIV-1-infected individuals following ART discontinuation^1–6^. A major gap in our knowledge is an understanding of the molecular and cellular origin of rebound virus. Prior studies from clinical trials of HIV-1-specific broadly neutralizing antibodies (bNAbs) in which individuals underwent analytical treatment interruption (ATI) reported that latent proviruses prior to ATI and rebound viruses following ATI share very limited overlapping *env* sequences, suggesting that rebound viruses are either not present or are rare in the viral reservoir^7–10^. Moreover, these studies suggested that rebound viruses represented recombinants of multiple latent proviruses found in the reservoir during ART suppression^7–10^. To evaluate this question in further detail, we generate near full-length sequences of putative replication-competent viral DNA in PBMC and LNMC from ART-suppressed, SIV-infected rhesus monkeys, and we compare these sequences to rebound viral RNA sequences following ART discontinuation. Here, we show that provirus in PBMC and LNMC is likely the direct origin of viral rebound in SIV-infected monkeys following ART discontinuation

## Results

**Fig. 1:**
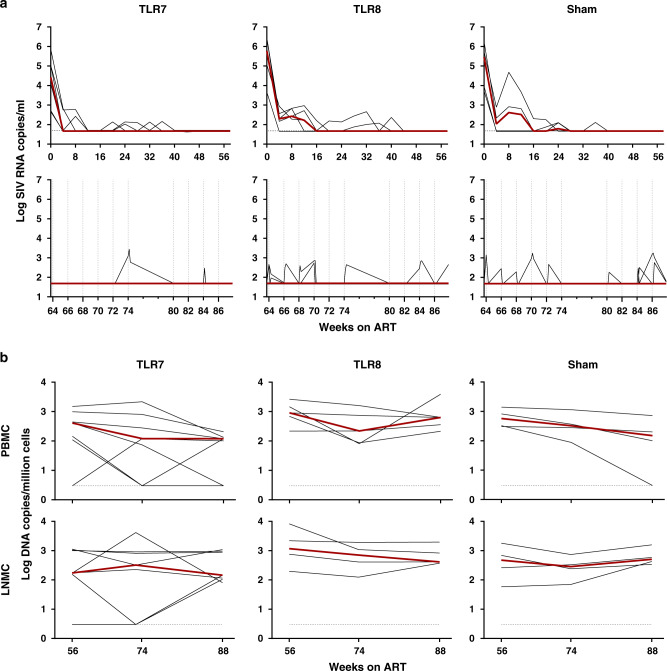
Plasma SIV RNA and cell-associated DNA during ART suppression. **a** Plasma viral RNA (log copies/ml) is shown before (weeks 0–64) and during (weeks 64–86) TLR administrations. Vertical dashed lines reflect the 10 TLR administrations. The *x*-axis represents weeks following ART initiation. **b** Cell-associated viral DNA (log copies/million cells) in PBMC and LNMC. Red lines indicate median values.

16 Indian-origin adult rhesus monkeys (*Macaca mulatta*) were chronically infected with SIVmac251 and were viremic for 85 weeks prior to ART initiation (Supplementary Fig. 1). At the time of ART initiation with TDF/FTC/DTG (week 0), monkeys had median plasma viral loads of 5.04 log RNA copies per ml (range 2.67–6.45 log RNA copies per ml) and were divided into three groups based on viral loads (Fig. 1a). Viral RNA declined following initiation of ART but animals with higher viral loads required up to 40 weeks for full virologic suppression. Residual viral blips were observed during ART in all groups. From week 64–86, 10 doses of TLR7 (GS-9620) or TLR8 (GS-566) agonists were administered to monkeys approximately every two weeks. Blood was collected on days 0, 1, 2 relative to each TLR agonist administration and was assessed for viral blips and evidence of cellular immune activation (Fig. 1a; Supplementary Fig. 2). Viral blips were observed in 2 monkeys in the TLR7 agonist group, 2 monkeys in the TLR8 agonist group, and 3 monkeys in the sham group, suggesting that the viral blips were not related to TLR7 or TLR8 agonist administration, consistent with a previous report^11^. Viral DNA levels remained relatively stable in the sham group over the course of the study, and there was no clear impact of either TLR7 or TLR8 agonists on viral DNA levels in PBMC or LNMC (Fig. 1b). Both TLR7 and TLR8 agonists led to activation of CD4+ T cells in PBMC as evidenced by increased CD69 expression by day 1 after TLR agonist administration (Supplementary Fig. 2a), as well as enhanced TLR7- and TLR8-specific plasma cytokine profiles (Supplementary Fig. 2b).

**Fig. 2:**
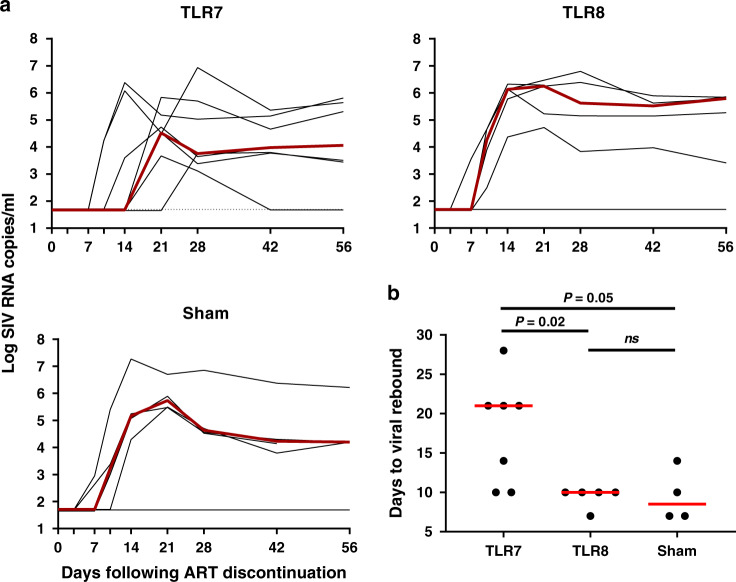
Plasma SIV RNA following after ART discontinuation. **a** Plasma viral RNA (log copies/ml) is shown for days 0–56 following ART discontinuation for the TLR7 agonist (*n* = 7), TLR8 agonist (*n* = 5), and sham groups (*n* = 4). **b** Days to viral rebound defined as the first detectable plasma SIV RNA in each animal. Red horizontal bars indicate median values. *P* values calculated using two-sided Mann–Whitney tests.

At week 90, ART was discontinued in all groups, and animals were monitored for viral rebound. Viral rebound in plasma was observed in all animals (Fig. 2a). All sham controls rebounded by day 7–10 following ART discontinuation and exhibited median setpoint plasma SIV RNA levels of 4.38 log copies per ml (range 4.29–6.42 log copies per ml). Animals in the TLR8 agonist group also rebounded by day 7–10 following ART discontinuation and exhibited median setpoint plasma SIV RNA levels of 5.71 log copies per ml (range 3.58–6.34 log copies per ml). In contrast, animals in the TLR7 agonist group rebounded by day 10–28 following ART discontinuation and exhibited median setpoint plasma SIV RNA levels of 3.89 log copies per ml (range 2.38–6.09 log copies per ml). Thus, the TLR7 agonist GS-9620 led to a modest 2.5-fold delay in the time to viral rebound from a median of 8.5 to 21 days, as compared with the TLR8 agonist group and the sham controls (*P* = 0.02 and *P* = 0.05, respectively, Mann–Whitney test) (Fig. 2; Supplementary Fig. 3), consistent with our previous published data^12,13^.

**Fig. 3:**
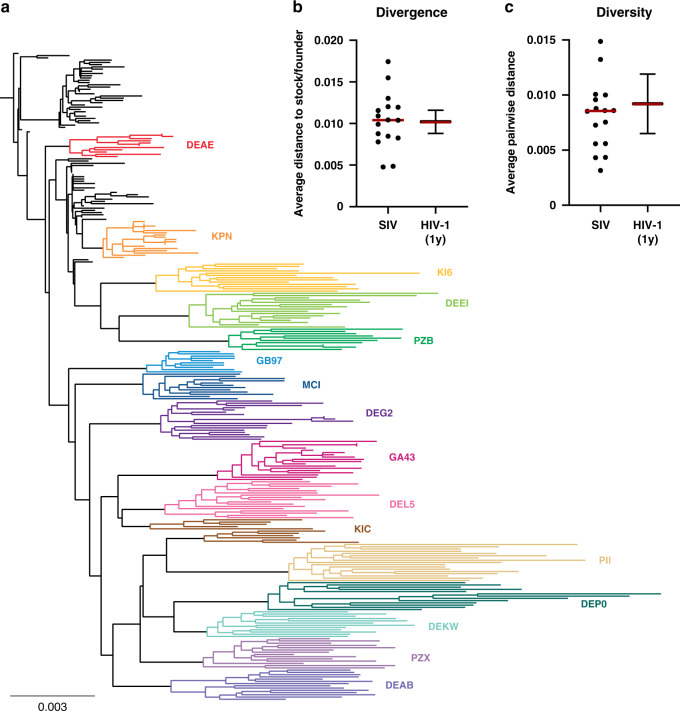
Diversity of plasma viral RNA sequences prior to ART initiation. **a** Phylogenetic tree of *env* sequences from plasma viral RNA in viremic, chronically SIV-infected monkeys at 14 weeks prior to initiation of ART. The black lines represent the SIVmac251 challenge stock and viral RNA sequences from each animal are shown in colors. **b** Average phylogenetic distance of plasma virus from each animal (*n* = 16) to the SIVmac251 challenge stock. **c** Average pairwise distance of virus population diversity for each animal (*n* = 16). **b** and **c** Viral divergence and viral diversity within each animal were compared to previously reported values of HIV-1-infected individuals after 1 year of infection^14^. Red horizontal bars indicate median values.

We utilized this cohort of animals to compare the sequences of rebound viral RNA following ART discontinuation, pre-ART viral RNA and near full-length viral DNA in PBMC prior to ART initiation, and near full-length viral DNA in PBMC and LNMC during ART suppression. Pre-ART viral RNA was extracted from plasma from viremic monkeys at week-14 prior to ART initiation, and 10–15 full-length *env* and *gag* + *pol* sequences were generated by single genome amplification (SGA). Sequences were aligned and compared to the reference SIVmac251 challenge stock, and neighbor-joining trees showed the viral diversity and divergence of circulating virus in these animals (Fig. 3a). Viral sequences isolated from each animal were clustered in distinct lineages that separated from other animals. The average distance of the sequences from each animal to the SIVmac251 challenge consensus ranged from 0.0048 to 0.0174 with the median of 0.0104 base substitutions per site (Fig. 3b), and the average pairwise distance of virus population diversity for each animal ranged from 0.0032 to 0.0149 with the median of 0.0086 base substitutions per site (Fig. 3c). The viral divergence to the stock and viral diversity within each animal were comparable to those parameters in HIV-1-infected individuals after 1 year of chronic infection (0.0102 ± 0.0014 and 0.0092 ± 0.0027 per year, respectively)^14^. These data demonstrate substantial viral sequence diversity in the chronically SIV-infected animals prior to ART initiation.

**Fig. 4:**
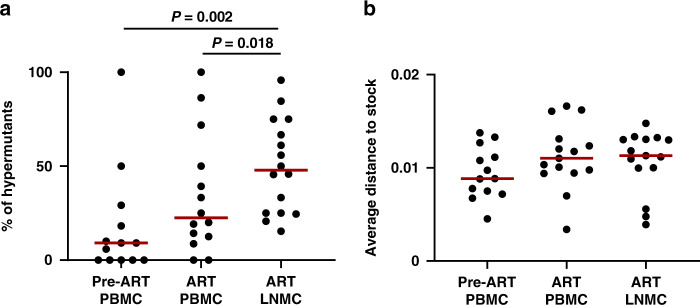
Near full-length (NFL) viral DNA from PBMC and LNMC. **a** Percentage of hypermutated NFL viral DNA sequences in each animal (*n* = 16). **b** Average distance of putative replication-competent NFL (rcNFL) viral DNA sequences in each animal (*n* = 16) compared to the SIVmac251 challenge stock. Viral DNA from PBMC prior to and during ART suppression, and from LNMC during ART suppression are shown. Red lines indicate median values (*n* = 16). *P* values calculated using two-sided Mann–Whitney tests.

We next sequenced near full-length (NFL) viral DNA prior to and during ART suppression. Genomic DNA was extracted from PBMC during chronic infection prior to ART initiation (Pre-ART PBMC), PBMC during ART suppression (ART PBMC), and LNMC during ART suppression (ART LNMC). A total of 800 NFL viral sequences were obtained (Supplementary Fig. 4). Similar to previous findings^15^, we observed that 9% of NFL viral DNA sequences in PBMC were hypermutated prior to ART initiation (Fig. 4a), and this percentage increased to 20% during ART suppression. Notably, the percentage of hypermutated NFL viral sequences was 52% in LNMC during ART suppression, suggesting potential anatomic differences in PBMC and LNMC viral reservoirs and indicating that over half of NFL viral genomes in lymph nodes were defective.

We established a pipeline to identify putative replication-competent NFL (rcNFL) viral DNA with intact *gag*, *pol*, and *env* open reading frames and Ψ/MSD sequences (Supplementary Fig. 5). A total of 500 rcNFL viral DNA sequences were obtained, and we selected the most diverse *env* gene for detailed analysis. The average distance of *env* sequences from rcNFL viral DNA to the consensus sequence of the SIVmac251 challenge stock was calculated (Fig. 4b). The phylogenetic distances of rcNFL viral DNA from pre-ART PBMC, ART PBMC, and ART LNMC samples to the stock were comparable, suggesting that there was no substantial viral evolution while on ART, consistent with previous studies^16–18^. In addition, identical rcNFL sequences were rare and were only identified in monkey KI6 and MCI (Supplementary Fig. 6), presumably due to the length of therapy as previous studies suggested^19,20^.

**Fig. 5:**
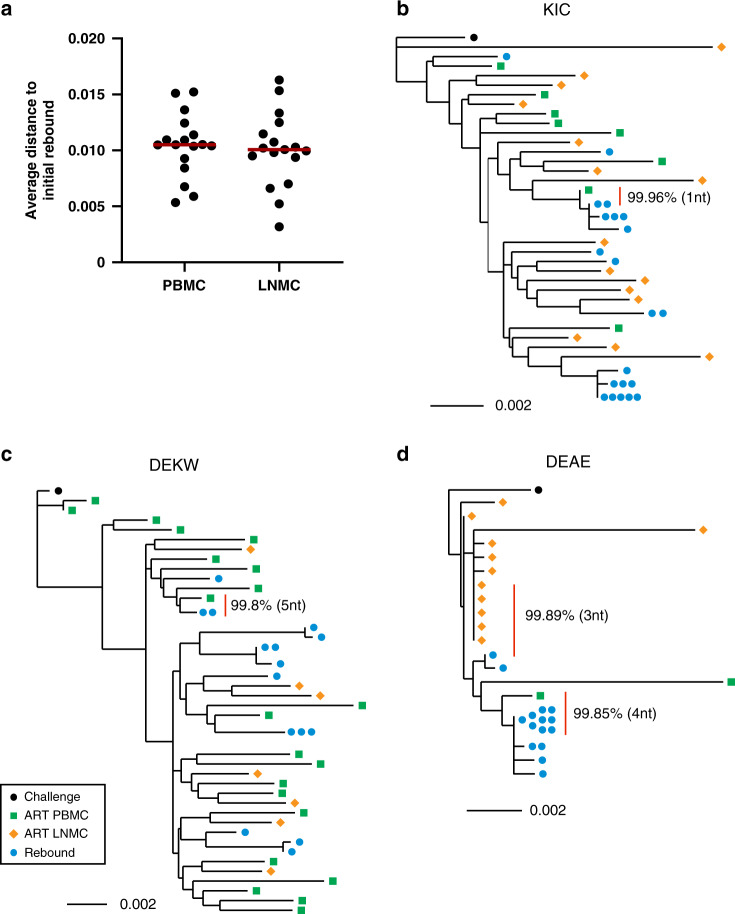
Initial rebound viral RNA sequences compared with rcNFL viral DNA sequences from PBMC and LNMC. **a** Average distance of *env* sequences from the initial rebound virus to rcNFL viral DNA in PBMC and LNMC in each animal (*n* = 16). **b** to **d** Phylogenetic trees of initial plasma rebound viral RNA sequences following ART discontinuation and rcNFL viral DNA sequences from PBMC and LNMC from three animals (KIC, DEKW, DEAE). The closest matches are indicated with a vertical red line with the sequence identity indicated.

Following ART discontinuation, viral loads were monitored twice weekly in all animals. To evaluate whether the rebound viral RNA sequences were genetically related to rcNFL viral DNA sequences from PBMC or LNMC during ART suppression, we isolated initial plasma rebound viruses in each monkey 1–4 weeks after ART discontinuation (Fig. 2), and *env* regions were sequenced. The median distance of rcNFL viral DNA in ART PBMC and ART LNMC to the initial rebound viral RNA was comparable (Fig. 5a). *env* sequences from the initial rebound viral RNA and rcNFL viral DNA during ART suppression were then aligned with the SIVmac251 challenge stock, and neighbor-joining trees were generated for all 16 monkeys (Fig. 5b–d; Supplementary Fig. 6).

The initial rebound viral RNA sequences closely clustered with putative rcNFL viral DNA sequences from PBMC and LNMC in all animals. We identified nearly identical sequences to the initial rebound viruses in the putative rcNFL viral DNA sequences in PBMC and LNMC during ART suppression. In monkey KIC (Fig. 5b), the closest initial rebound viral RNA sequences matched rcNFL viral DNA sequences in PBMC by 99.96% (1 nucleotide difference). Within the same rebound viral lineage, additional rebound viral RNA sequences matched rcNFL viral DNA sequences in PBMC by 99.92% and 99.84% (2 and 4 nucleotide difference), respectively. In monkey DEKW (Fig. 5c), the initial rebound viral RNA sequences matched rcNFL viral DNA sequences in PBMC by 99.81% (5 nucleotide difference). In monkey DEAE (Fig. 5d), the initial rebound viral RNA sequences matched rcNFL viral DNA sequences in LNMC by 99.89% (3 nucleotide difference) and in PBMC by 99.85% (4 nucleotide difference). For the remaining 13 animals (Supplementary Fig. 6), the initial rebound viral RNA sequences matched rcNFL viral DNA sequences by 99.2–99.8%, except for monkey DEL5 that likely had insufficient samples for sequencing but still showed a 98.3% match. Similar results were obtained by including Pre-ART PBMC sequences in the trees (Supplementary Fig. 7). These data demonstrate that putative rebound-competent proviral DNA persisted in PBMC and LNMC despite prolonged ART suppression and closely matched the initial rebound virus following ART discontinuation.

**Fig. 6:**
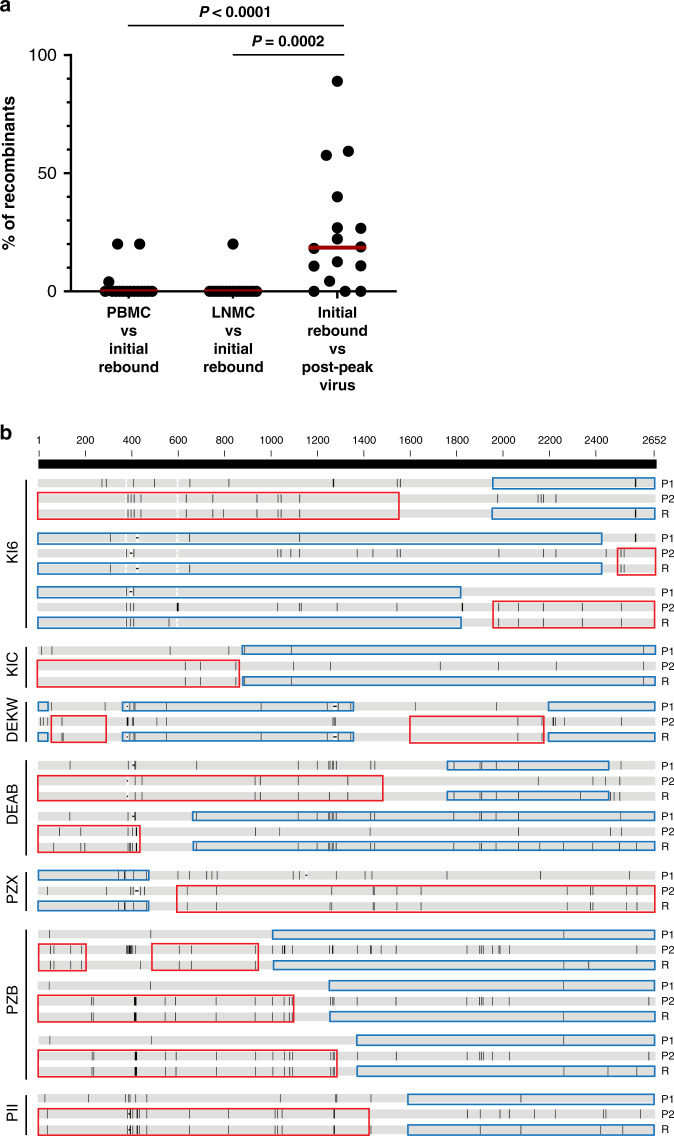
Recombination events in rebound viruses. **a** Percentage of unique recombinant sequences in initial rebound virus compared to rcNFL viral DNA from PBMC and LNMC, and percentage of recombinant sequences in post-peak rebound virus compared to initial rebound virus. Red lines indicate median values (*n* = 16). *P* values calculated using two-sided Mann–Whitney tests. **b** Post-peak recombinant *env* sequences identified by RAPR program (LANL database) from seven representative animals. Recombinants with the *P* values less than 0.0001 from the Wald-Wolfowitz test were shown here. Red and blue rectangles showed the similar regions of the recombinants (R) and the parental sequences (P1 and P2).

To explore the degree of recombination in rebound viruses in the SIV-infected rhesus monkeys after ART discontinuation, we utilized a recently developed Recombination Analysis PRogram (RAPR, LANL database)^21^. We first analyzed the extent of recombination in the initial rebound viruses compared with putative rcNFL viral DNA sequences in PBMC or LNMC. We observed recombination events in the initial rebound viruses in 3 of 16 animals compared with rcNFL viral DNA in PBMC and in 1 of 16 animals compared with rcNFL viral DNA in LNMC in a minority (<20%) of all sequences (Fig. 6a). We next sequenced viral RNA at a later timepoint reflecting post-peak viremia, which represented 2–4 weeks after initial viral rebound (Fig. 2). In contrast to the initial rebound viruses, 13 of 16 monkeys demonstrated recombination at this timepoint in 5–83% of all sequences (*P* < 0.0001 and *P* = 0.0002 compared with recombination of initial viral rebound sequences with rcNFL viral DNA in PBMC and LNMC, respectively; Fig. 6a and Supplementary Fig. 8). To evaluate the detailed recombinant structures in post-peak viral RNA sequences, we analyzed recombination breakpoints. The post-peak rebound recombinants with statistically significant (*P* < 0.001) breakpoint intervals typically included one or more initial rebound virus sequences (Fig. 6b). Moreover, we isolated post-peak rcNFL viral DNA sequences in PBMC in 3 animals and compared to rebound virus (Supplementary Fig. 9). In monkey KIC, we identified 2 recombinants that were only one nucleotide different from rcNFL viral DNA sequences in PBMC, suggesting that post-peak recombinants were derived from PBMCs that were not previously identified.

## Discussion

Our data demonstrate that initial rebound viruses were largely non-recombinant and closely match putative rcNFL viral DNA sequences in the viral reservoir in SIV-infected rhesus monkeys. Recombinant viruses emerged quickly after viral rebound after treatment discontinuation but were rare in initial rebound virus populations. These findings are consistent with a prior report showing that rebound viruses were heterogeneous across different tissues^22^. However, these findings contrast with recent studies that claimed that rebound viruses in HIV-1-infected humans following discontinuation of ART consisted primarily of recombinants of latent proviruses, suggesting that individual proviruses in the latent reservoir were not the direct origin of rebound virus^7–10^. One possibility is that HIV-1-infected humans and SIV-infected rhesus monkeys are fundamentally different in this regard^15,23,24^. Another possibility is that the true initial rebound viruses were not obtained in the clinical studies as a result of insufficient sampling frequency. Indeed, our data show that recombinant viruses emerge quickly in almost all animals within 2–4 weeks after initial rebound, and probably much earlier (Fig. 6a). It is also possible that the rare low frequency recombinants detected in the initial rebound viruses in our SIV-infected rhesus monkeys also reflect suboptimal sampling frequency. Finally, it is possible that additional depth of sequencing may be required in chronically HIV-1-infected individuals with substantial sequence diversity in the viral reservoir. The SIV diversity and divergence in our chronically SIV-infected animals were similar to HIV-1 after one year of infection (Fig. 3) but still less than HIV-1 after many years of infection^14^.

The sequence match between the initial rebound virus and the putative rcNFL viral DNA was >99% in 15 of 16 animals and was >99.5% in 9 of 16 animals. Given the tremendous viral diversity, one would not expect to find the exact match of the rebound virus in the reservoir unless the rebounding virus originated from a highly expanded clone or greater sequencing depth can be achieved. Due to sample availability, the depth of rcNFL sequencing was limited in this study. On the other hand, consistent with a previous study^15^, we observed that identical viral DNA sequences from a single animal were rare in SIV-infected monkeys with 1–2 years of infection and/or treatment.

In summary, we evaluated the putative origin of rebound virus following ART discontinuation in the well-controlled model of ART-suppressed, chronically SIV-infected rhesus monkeys. By fine monitoring of the kinetics of viral rebound after ART discontinuation, we observed that initial rebound viral RNA sequences closely matched putative rcNFL viral DNA sequences in PBMC and LNMC during ART suppression. These data strongly suggest that rebound viruses emerged directly from individual cells in the viral reservoir, without the need for viral recombination. Recombination nevertheless occurred frequently and quickly following rebound in the context of active viral replication, as expected, but initial plasma rebound viruses were generally non-recombinant. These data provide fundamental insights into reservoir biology and critically focus HIV-1 cure efforts on the rebound-competent intact viral reservoir.

## Methods

### Monkeys and study design

Sixteen outbred, Indian-origin adult male and female rhesus monkeys (Macaca mulatta) were genotyped and selected as negative for the protective major histocompatibility complex (MHC) class I alleles Mamu-A*01, Mamu-B*08, and Mamu-B*17. Tripartite motif-containing protein 5 (TRIM5) polymorphisms were balanced equally among groups. All monkeys were housed at Alpha Genesis, Yemassee, SC. Animals were infected with IR SIVmac251 at week-85. Plasma viral RNA was isolated at week-14 prior to ART initiation for viral diversity and divergence analysis. Following infection, all the monkeys were viremic until ART initiation at week 0. ART was administered daily by subcutaneous injection with a pre-formulated cocktail of tenofovir, emtricitabine, and dolutegravir (Gilead, Foster City, CA) for 64 weeks prior to initiation of TLR administration. Monkeys were divided into three groups based on viral loads at the time of ART initiation. Group I (*N* = 7) received either 0.15 mg/kg or 0.5 mg/kg TLR7 agonist GS-9620 (Gilead), Group II (*N* = 5) received TLR8 agonist GS-566 (Gilead) at 6 mg/kg for doses 1 to 5 and at 10 mg/kg for doses 6 to 10, and Group III (*N* = 4) was the sham control. TLR agonists were administrated every two weeks for a total of ten doses, with a break of six weeks between 6th and 7th dose. Blood was collected on day 0, 1, and 2 after each TLR administration. At week 90, ART was discontinued in all groups to monitor for viral rebound. Plasma was collected twice weekly to monitor viral rebound. For viral sequencing, pre-ART plasma and PBMC were collected at week-14, ART PBMC was collected at weeks 40–58, ART LNMC was collected at weeks 56–64. Immunologic and virologic assays were performed blinded. Monkeys were maintained according to the guidelines of the NIH Guide to the Care and Use of Laboratory Animals. All monkey studies were approved by the Alpha Genesis Institutional Animal Care and Use Committee (IACUC).

### Viral RNA assays

Viral RNA was isolated from cell-free plasma using a viral RNA extraction kit (Qiagen) and was quantified essentially as described^13^.

### Viral DNA assays

Viral DNA was quantified as previously described^13^. Briefly, total cellular DNA was isolated from 5 million cells using a QIAamp DNA Blood Mini kit (Qiagen). The absolute quantification of viral DNA in each sample was determined by qPCR using primers specific to a conserved region SIVmac239. All samples were directly compared to a linear virus standard and the simultaneous amplification of a fragment of human RPP30 gene. PCR assays were performed with 100–200 ng sample DNA.

### Plasma cytokine analysis

EDTA whole blood was collected pre-administration and post-administration of the TLR7 and TLR8 agonists at each dose. Cytokine and chemokine measurements were performed on plasma by Luminex assay on Luminex 200 system using a 29-plex monkey panel per manufacturer’s instructions (ThermoFisher Scientific). Data was analyzed by subtracting the pre-dose value from the post-dose value for each analyte at each dose for each animal and a mean was calculated for each dose. Data were plotted using GraphPad Prism (GraphPad Software).

### Single genome amplification

Single-genome amplification SGA assays were performed essentially as described^25^. Briefly, viral RNA was isolated and reverse transcribed to viral cDNA using SIV-Env-OutR (5′-TGTAATAAATCCCTTCCAGTCCCCCC-3′). First-round PCR was carried out with Q5 High-Fidelity 2× Master Mix (NEB) together with primer SIV-Env-OutF (5′-CCTCCCCCTCCAGGACTAGC-3′). PCR conditions were programmed as follows, 1 cycle of 98 °C for 30 s, 35 cycles of 98 °C for 15 s, 55 °C for 15 s and 72 °C for 2 min, followed by a final extension of 72 °C for 10 min. 1 μL of first-round PCR product was add to Q5 2× Master Mix with primer SIV-Env-InnF (5′-ATAGACATGGAGACACCCTTGAGGGAGC-3′) and SIV-Env-InnR (5′-ATGAGACATRTCTATTGCCAATTTGTA-3′). PCR conditions were programmed as above but increased to 45 cycles for the second step. Amplicons from cDNA dilutions resulting in less than 30% positive were considered to result from amplification of a single cDNA amplification and were processed for sequencing. For each sample, 15–30 sequences were analyzed. Sanger sequencing was performed by DF/HCC DNA Resource Core (Boston, MA) and the sequences were assembled and analyzed by Sequencher (Gene Codes Corporation).

### Near full-length viral sequencing

Genomic DNA was isolated from PBMC of monkeys at week −14 (Pre-ART PBMC), as well as from PBMC at weeks 40–58 and LNMC at weeks 56–64 during ART suppression (ART PBMC and ART LNMC). Size selection by Blue pippin (Sage Science) was performed according to the manufacturer’s protocol to obtain the DNA fragments containing potential NFL viral DNA, followed by a limiting-dilution semi-nested PCR. PCR was carried out with LongAmp Taq DNA Polymerase (New England Biolabs) together with primers SIV-NFL-OutF (5′-TAGCAGATTGGCGCCCGAA-3′) and SIV-NFL-OutR (5′-CCCTTCCAGTCCCCCCTTTTCTTT-3′) for first-round PCR, and SIV-NFL-InnF (5′-CTATAAAGGCGCGGGTCGGTA-3′) and SIV-NFL-InnR (5′-GCTCTTAGGGGAACTTTTGGCC-3′) for second-round PCR. PCR conditions were programmed a. Amplicons from dilutions resulting in less than 30% positive were considered to result from amplification of a single viral amplification and were processed for sequencing. For each sample, 15 to 30 sequences were analyzed. Viral genome sequencing was performed by the CCIB DNA Core Facility at Massachusetts General Hospital (Cambridge, MA). The raw sequencing data were analyzed by quality control pipelines only to obtain the viral genomes without any detectable deletions. A total of 800 intact NFL sequences were obtained. Hypermutated NFL sequences were analyzed by the Hypermut 2.0 (www.hiv.lanl.gov) and by manually check. The identified hypermutants were excluded for downstream replication competency analysis. The remaining sequences were further checked by a pipeline (Supplementary Fig. 4) to only obtain the rcNFL viral sequences with intact open-reading frames of major viral transcripts and without pre-mature stop codons.

### Phylogenetic analysis

All alignments were made with GeneCutter (www.hiv.lanl.gov) and *env* regions were extracted for following analysis. A consensus sequence of SIVmac251 challenge stock was generated and used in all subsequent alignments^25^. The alignment was manually checked with Geneious Prime (Biomatters Ltd) to exclude any sequence with deletions or stop codons due to hypermutation. Neighbor-joining trees were constructed using the Kimura 2-parameter model with 1000 bootstrap replications (MEGA X)^26^. The consensus sequence of SIVmac251 challenge stock was used as the outgroup to root the trees. Viral diversity among lineages was measured as the mean pairwise distances as previously described^14^. Viral nucleotide change over time was measured as the mean distance of each taxa from the consensus sequence of challenge stock (MEGA X). All sequences have been deposited in GenBank with accession numbers MN884181-MN88543 and MW004671-MW004840.

### Recombination

*env* sequences were processed through ElimDupes program (www.hiv.lanl.gov) to eliminate sequences with identity larger than 99.9%. The resulting unique sequences were used as the baseline to compare to other timepoints by recombinant analysis program (RAPR) (www.hiv.lanl.gov)^21^. Recombinations were checked manually by highlighter plots and sequence alignments. False discovery rate (FDR) cut was adjusted accordingly (0.1–0.15) depending on the animals in order to include the recombinants that were identified manually. The percentage of recombination was calculated by the numbers of recombined sequences of total sequences.

### Statistical analyses

Analysis of virologic and immunologic data was performed using GraphPad Prism (GraphPad Software). Comparisons of groups was performed using two-sided Mann–Whitney tests without Bonferroni adjustments. Analysis of viral rebound was performed using Kruskal–Wallis tests to compare all groups. For group pairwise comparisons, area under the curve (AUC) for total viral RNA following ART discontinuation was compared with chi-square tests, and viral rebound was compared with a censored Poisson regression model. Except as stated, the experiments were randomized, and assays were performed blinded.

### Reporting summary

Further information on research design is available in the Nature Research Reporting Summary linked to this article.

## Supplementary information

Supplementary Information

Reporting Summary

## Data Availability

Supplementary Information and source data are available for this paper. All data generated and analyzed in this study are available from the corresponding author upon reasonable request. LANL Database tools were used for sequence analysis in this study (www.hiv.lanl.gov). All sequences have been deposited in GenBank with accession numbers MN884181-MN88543 and MW004671-MW004840 (https://www.ncbi.nlm.nih.gov/nuccore/). Source data are provided with this paper.

## Source data

Source Data
