# Supplementary material for: Origin of rebound virus in chronically SIV-infected Rhesus monkeys following treatment discontinuation
Source: Nat Commun. 2020 Oct 27;11:5412. doi: 10.1038/s41467-020-19254-2 (PMC7591481; doi:10.1038/s41467-020-19254-2)
Supplement: Supplementary file 1 — Supplementary Information [file 41467_2020_19254_MOESM1_ESM.pdf]

Origin of Rebound Virus in Chronically SIV-Infected Rhesus Monkeys Following Treatment Discontinuation
Supplementary Information

a

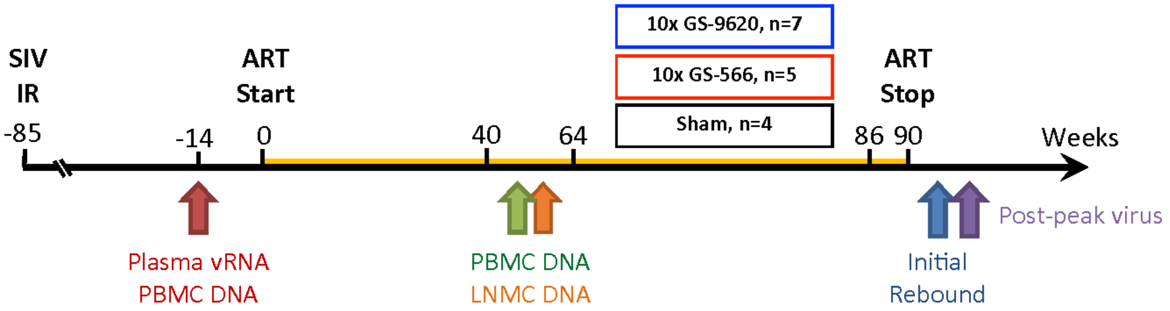

b

	Animal ID	Dose (mg/kg)
Group 1 TLR7 agonist (GS-9620)	1. KPN	0.15 mg/kg
	2. GA43	
	3. DEAE	
	4. KI6	
	5. MCI	0.5 mg/kg
	6. DEEI	
	7. DEL5	
Group 2 TLR8 agonist (GS-566)	8. DEG2	Dose 1-5: 6 mg/kg<br>Dose 6-10: 10 mg/kg
	9. PII	
	10. PZB	
	11. PZX	
	12. DEAB	
Group 3 Sham	13. DEKW	
	14. DEPO	
	15. KIC	
	16. GB97	

Supplementary Figure 1. Study design. a Sixteen rhesus monkeys were infected with SIVmac251 at week -85 and were viremic until ART initiation at week 0. TLR7 agonist (GS-9620) and TLR8 agonist (GS-566) were administered 10 times from weeks 64 to 86. ART was discontinued at week 90. Colored arrows showed the timepoints when plasma viral RNA or viral DNA was sequenced. **b** Animal IDs in each group.

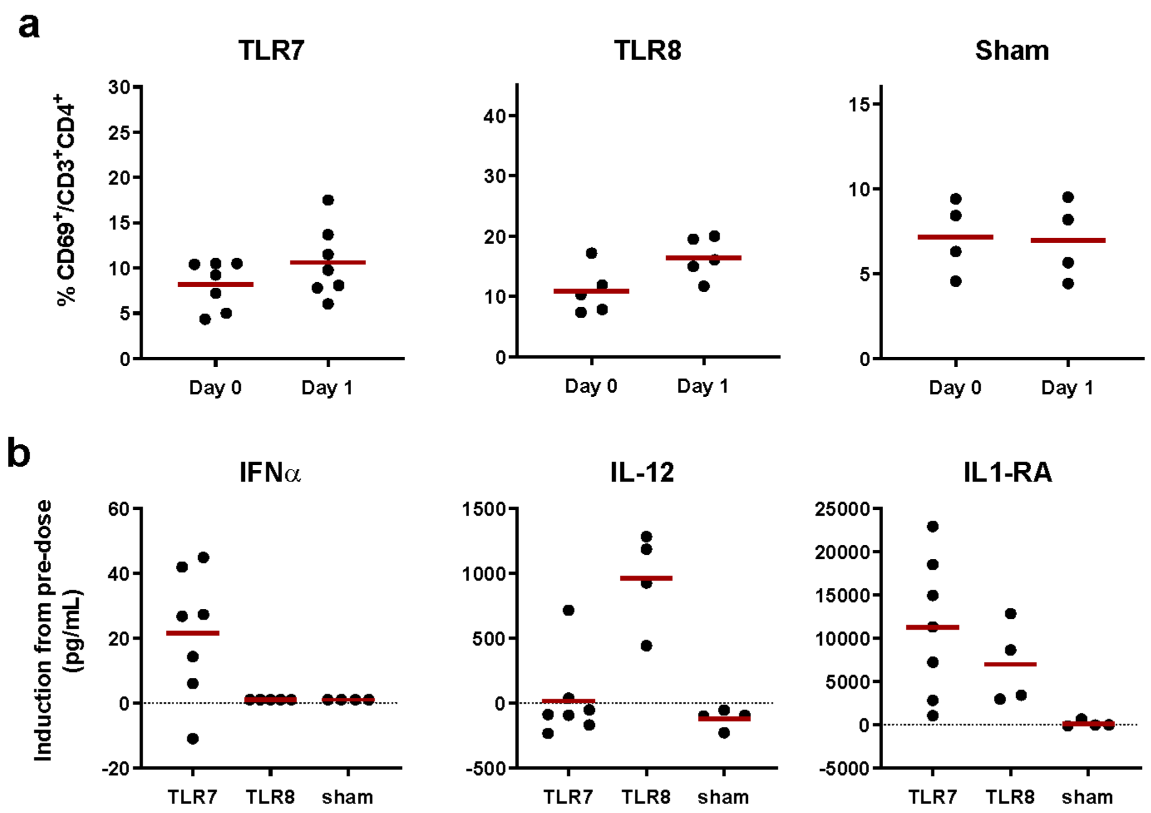

Supplementary Figure 2. Immune activation and plasma cytokine profile following TLR agonist administration. a Activation of CD4+ T cells was assessed by CD69 expression on days 0 and 1 following TLR agonist administration in the TLR7 agonist (n=7), TLR8 agonist (n=5), and sham groups (n=4). **b** Plasma cytokine following TLR agonist administration in the TLR7 agonist (n=7), TLR8 agonist (n=5), and sham groups (n=4). Values were shown by subtracting the pre-dose value from the post-dose value. Representative data are shown following the first TLR agonist dose, which were comparable to the subsequent TLR7 agonist doses. Red horizontal bars indicate mean values.

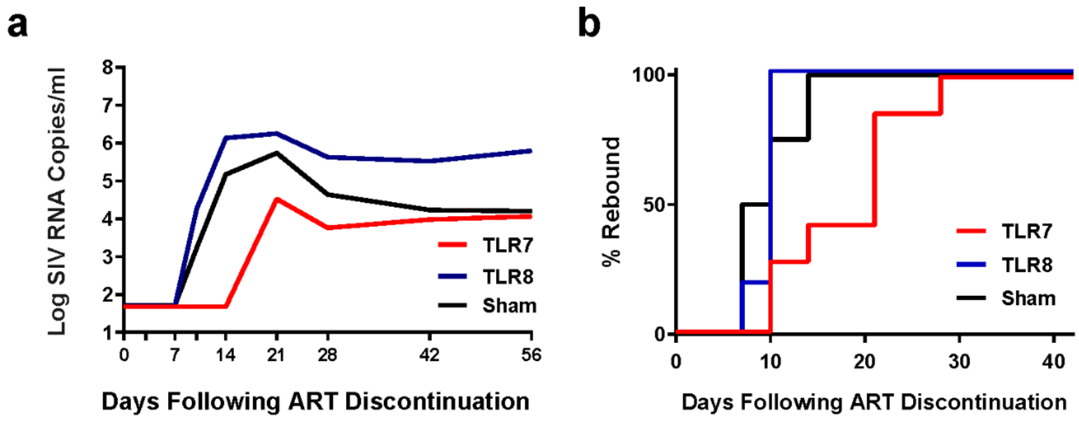

Supplementary Figure 3. Kinetics of viral rebound after treatment discontinuation. a Summary of median viral rebound kinetics in each group. Data are shown as median log SIV RNA copies/ml. **b** Kaplan–Meier curve shows the time to viral rebound. (A) and (B) n=7 in the TLR7 agonist group, n=5 in the TLR8 agonist group, and n=4 in the sham group.

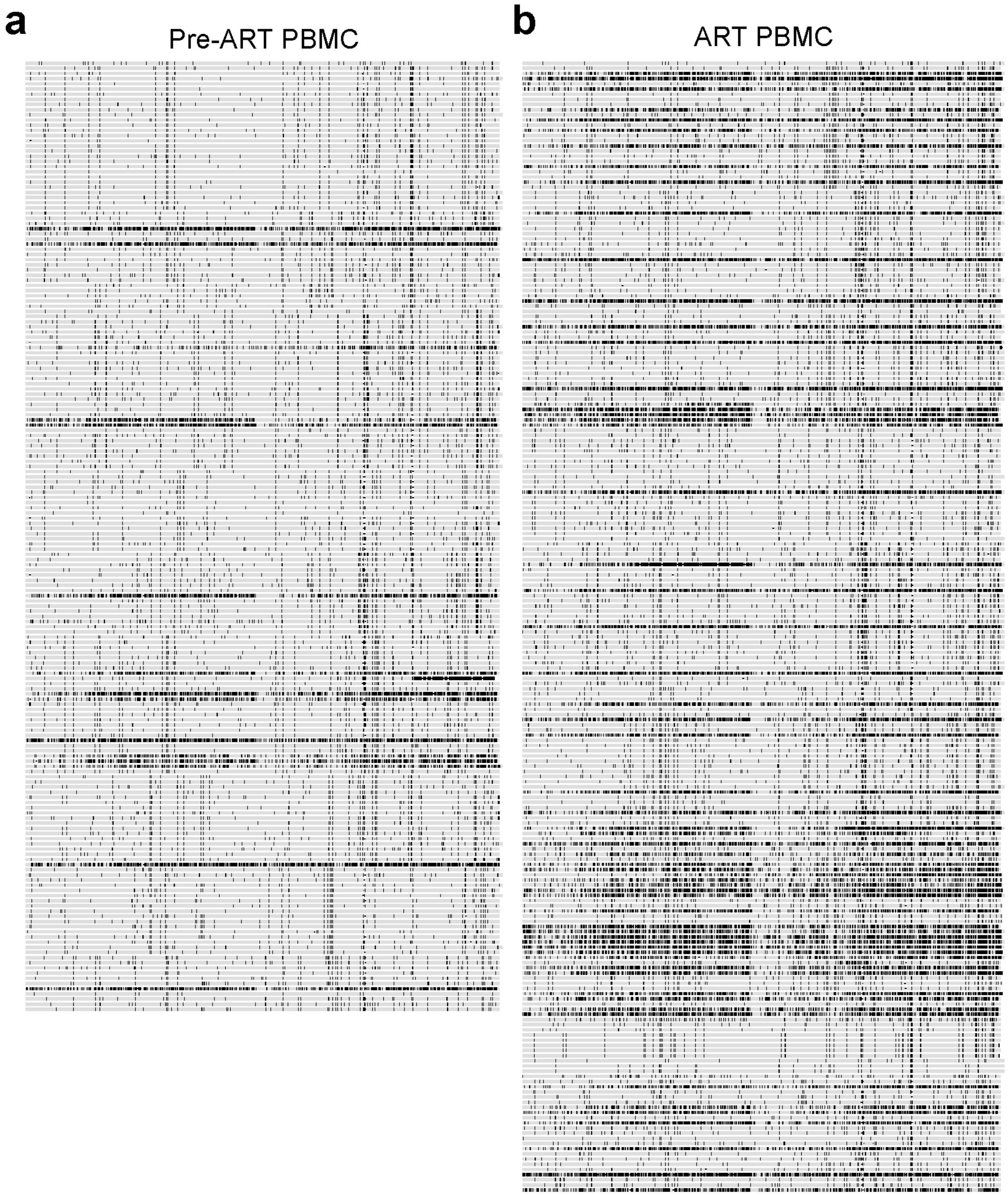
a
Pre-ART PBMC
b
ART PBMC

c

ART LNMC

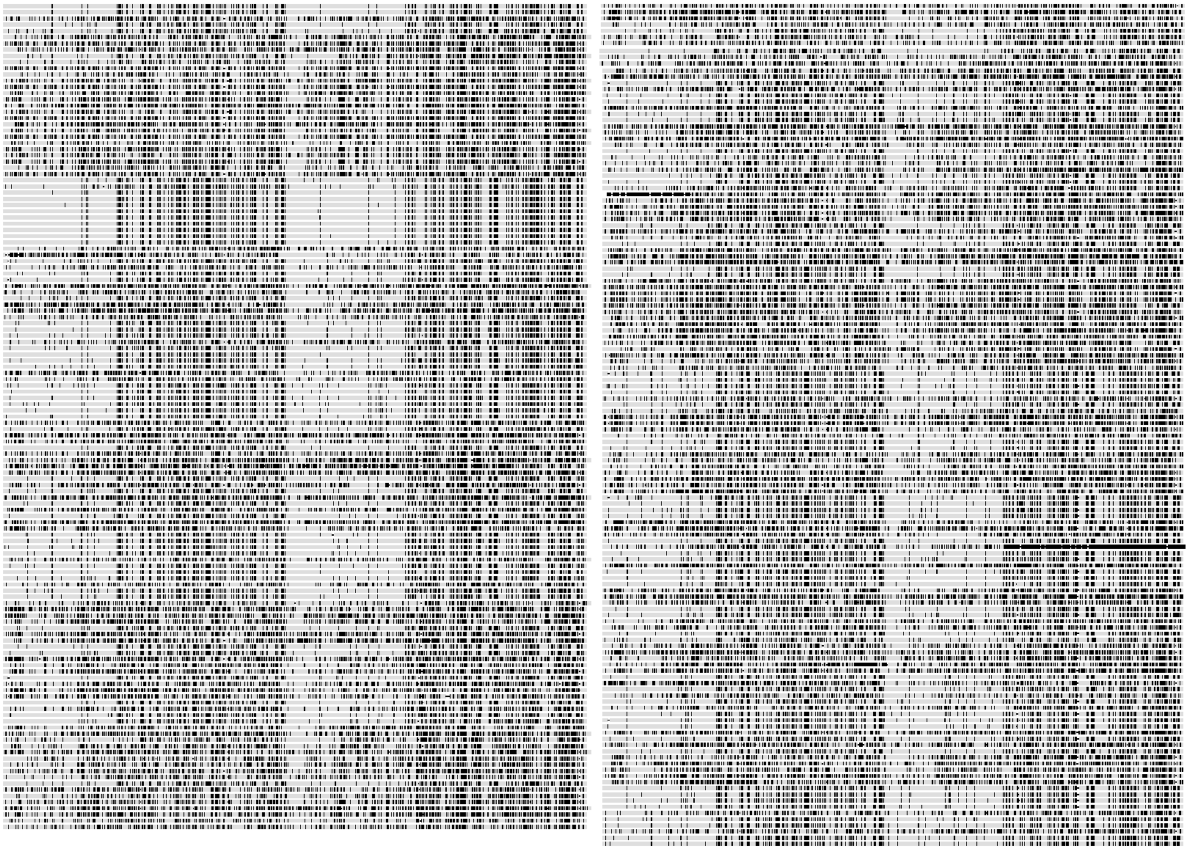

Supplementary Figure 4. NFL viral landscape in PBMCs and LNMCs. All NFL sequences harvested from **a** Pre-ART PBMC **b** ART PBMC and **c** ART LNMC samples were aligned to the SIVmac251 challenge stock consensus sequence. Individual horizontal lines represent 800 unique NFL sequences. Vertical black bars showed the nucleotide mismatches comparing to challenge stock consensus sequence.

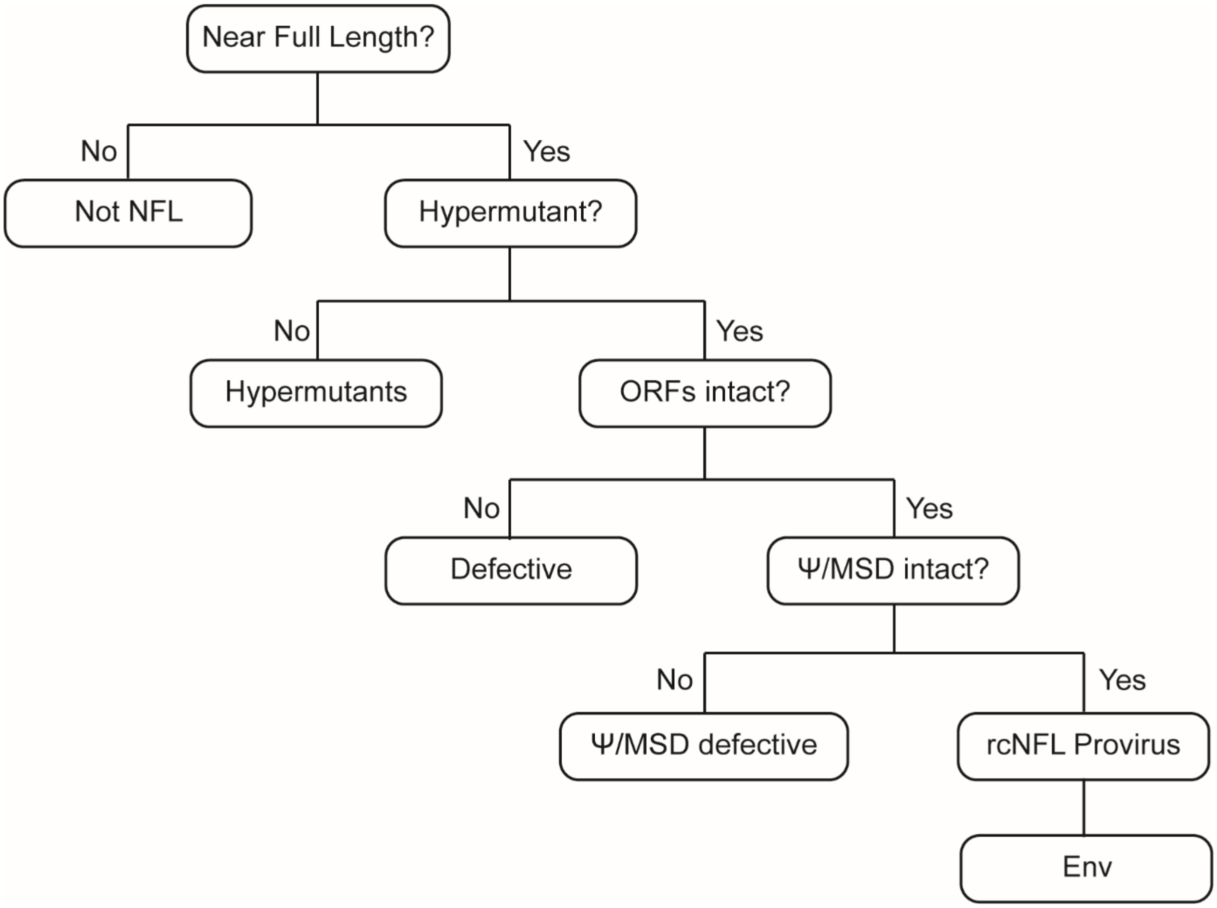

Supplementary Figure 5. Pipeline for identification of putative replication-competent NFL (rcNFL) viral DNA. Raw sequences were first filtered to obtain NFL viral DNA sequences at appropriate length. NFL sequences were analyzed by the Hypermut 2.0 (www.hiv.lanl.gov) and manually to exclude hypermutants that contain early stop codons in *gag*, *pol* or *env* regions. The remaining sequences that contained Ψ/MSD were considered as putative replication-competent NFL (rcNFL) viral DNA.

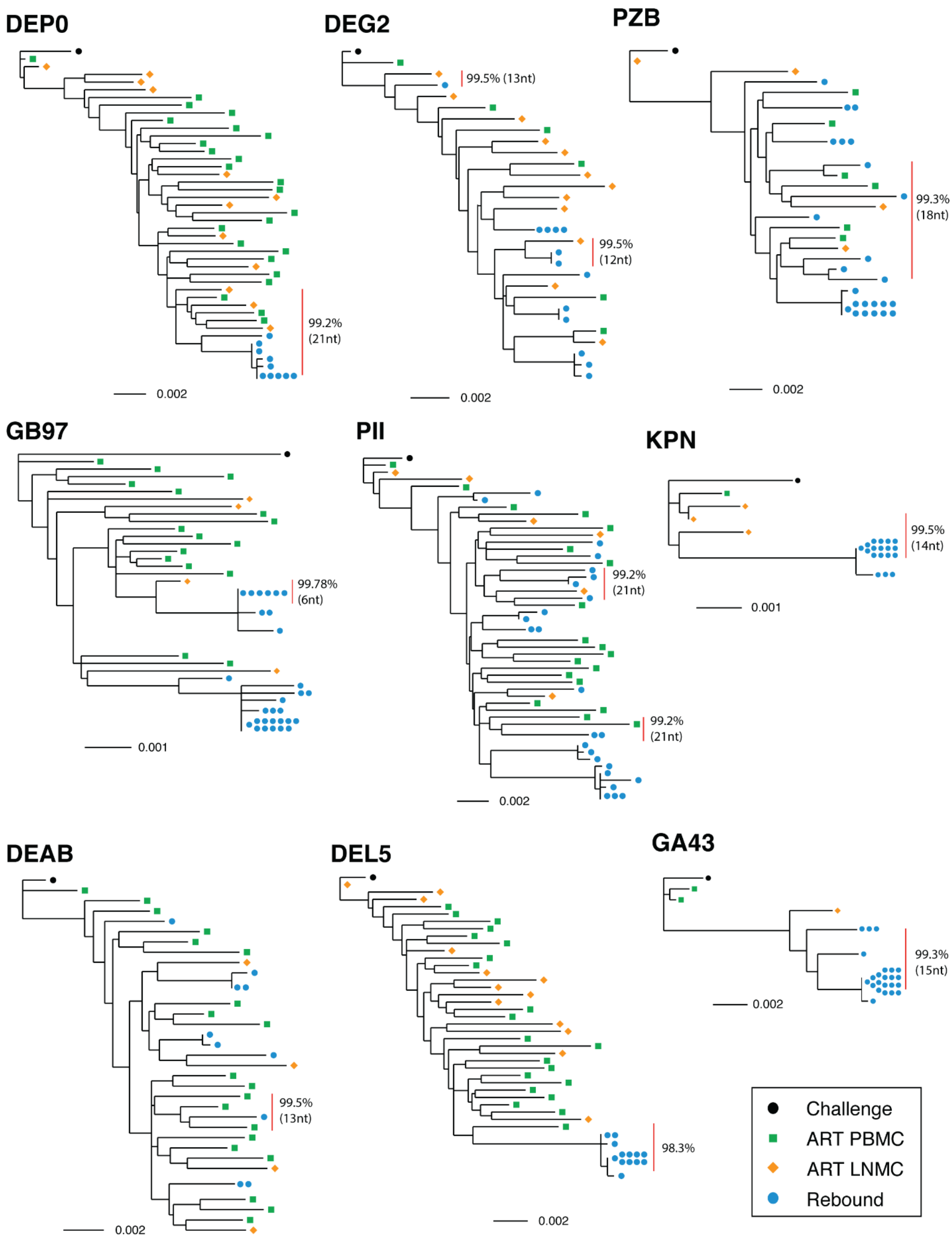
DEP0
99.2% (21nt)
0.002
DEG2
99.5% (13nt)
99.5% (12nt)
0.002
PZB
99.3% (18nt)
0.002
GB97
99.78% (6nt)
0.001
PII
99.2% (21nt)
99.2% (21nt)
0.002
KPN
99.5% (14nt)
0.001
DEAB
99.5% (13nt)
0.002
DEL5
98.3%
0.002
GA43
99.3% (15nt)
0.002
Challenge
ART PBMC
ART LNMC
Rebound

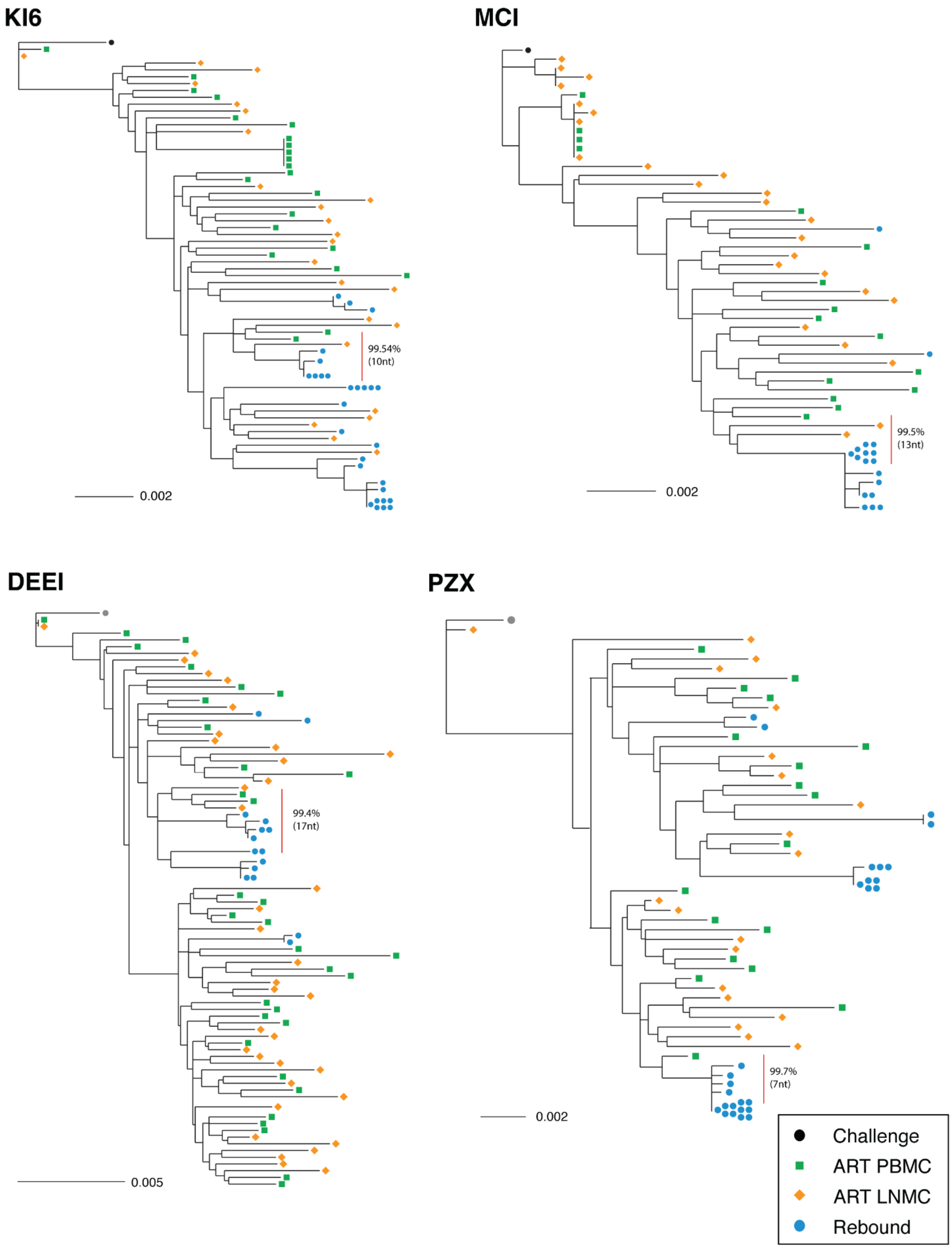

KI6
99.54%
(10nt)
0.002
MCI
99.5%
(13nt)
0.002
DEEI
99.4%
(17nt)
0.005
PZX
99.7%
(7nt)
0.002
Challenge
ART PBMC
ART LNMC
Rebound

Supplementary Figure 6. **Initial rebound viral RNA sequences compared with rcNFL viral DNA sequences from PBMC and LNMC.** Phylogenetic trees of initial plasma rebound viral RNA sequences following ART discontinuation and rcNFL viral DNA sequences from PBMC and LNMC from 13 animals. The closest matches are indicated with a vertical red line with the sequence identity indicated.

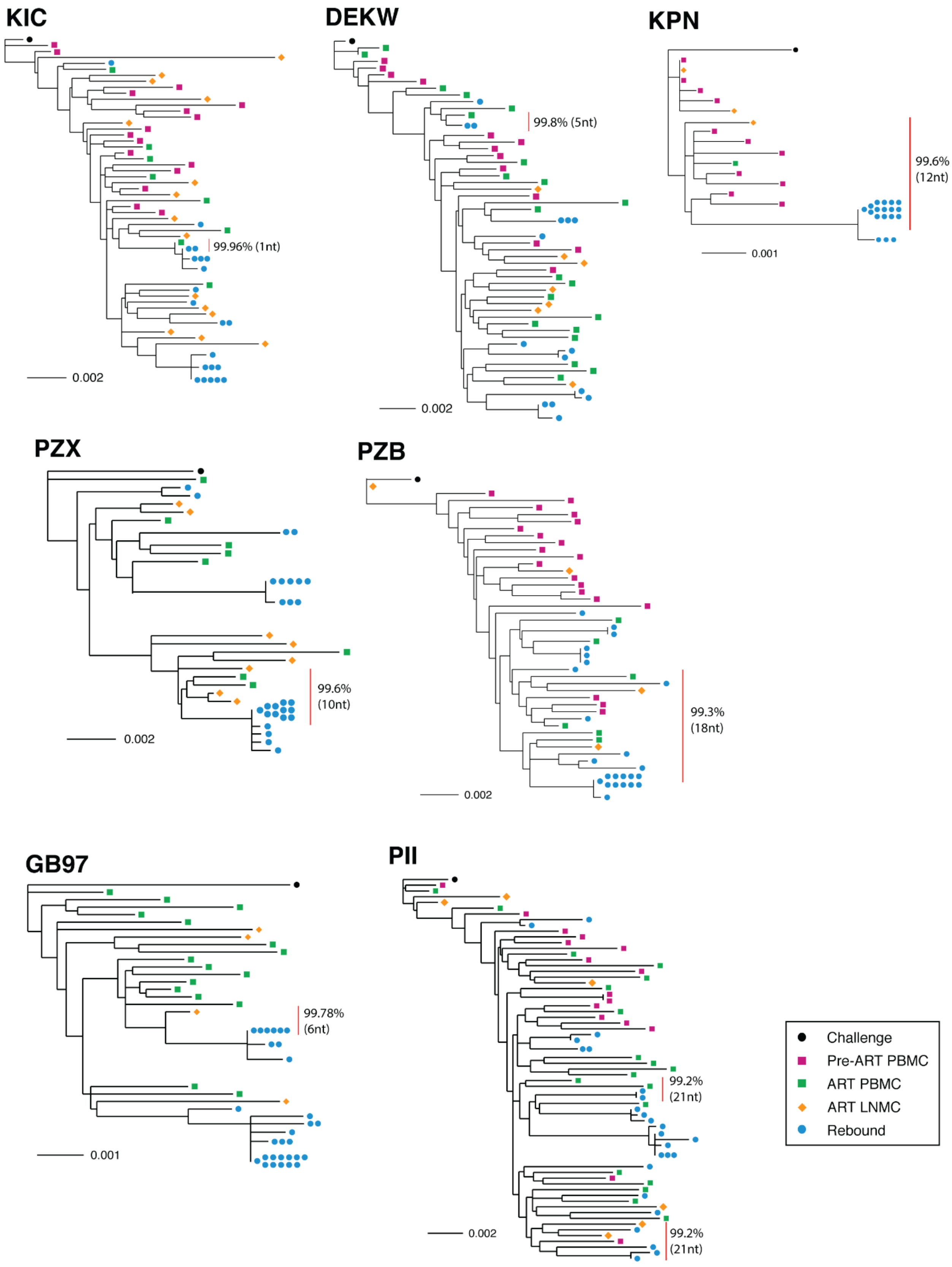
KIC
99.96% (1nt)
0.002
DEKW
99.8% (5nt)
0.002
KPN
99.6%
(12nt)
0.001
PZX
99.6%
(10nt)
0.002
PZB
99.3%
(18nt)
0.002
GB97
99.78%
(6nt)
0.001
PII
99.2%
(21nt)
99.2%
(21nt)
0.002
Challenge
Pre-ART PBMC
ART PBMC
ART LNMC
Rebound

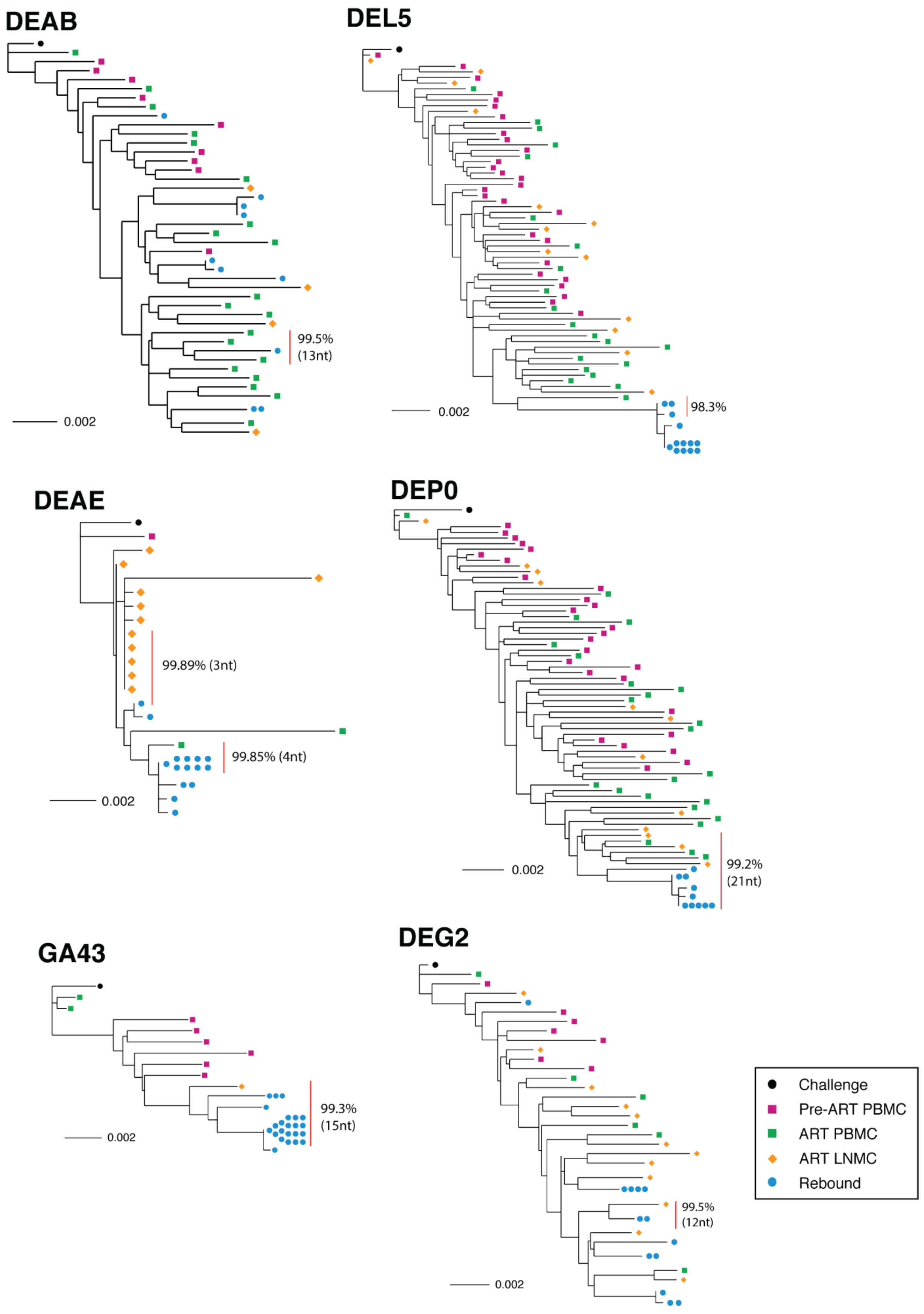

DEAB
99.5%
(13nt)
0.002
DEL5
98.3%
0.002
DEAE
99.89% (3nt)
99.85% (4nt)
0.002
DEP0
99.2%
(21nt)
0.002
GA43
99.3%
(15nt)
0.002
DEG2
99.5%
(12nt)
0.002
Challenge
Pre-ART PBMC
ART PBMC
ART LNMC
Rebound

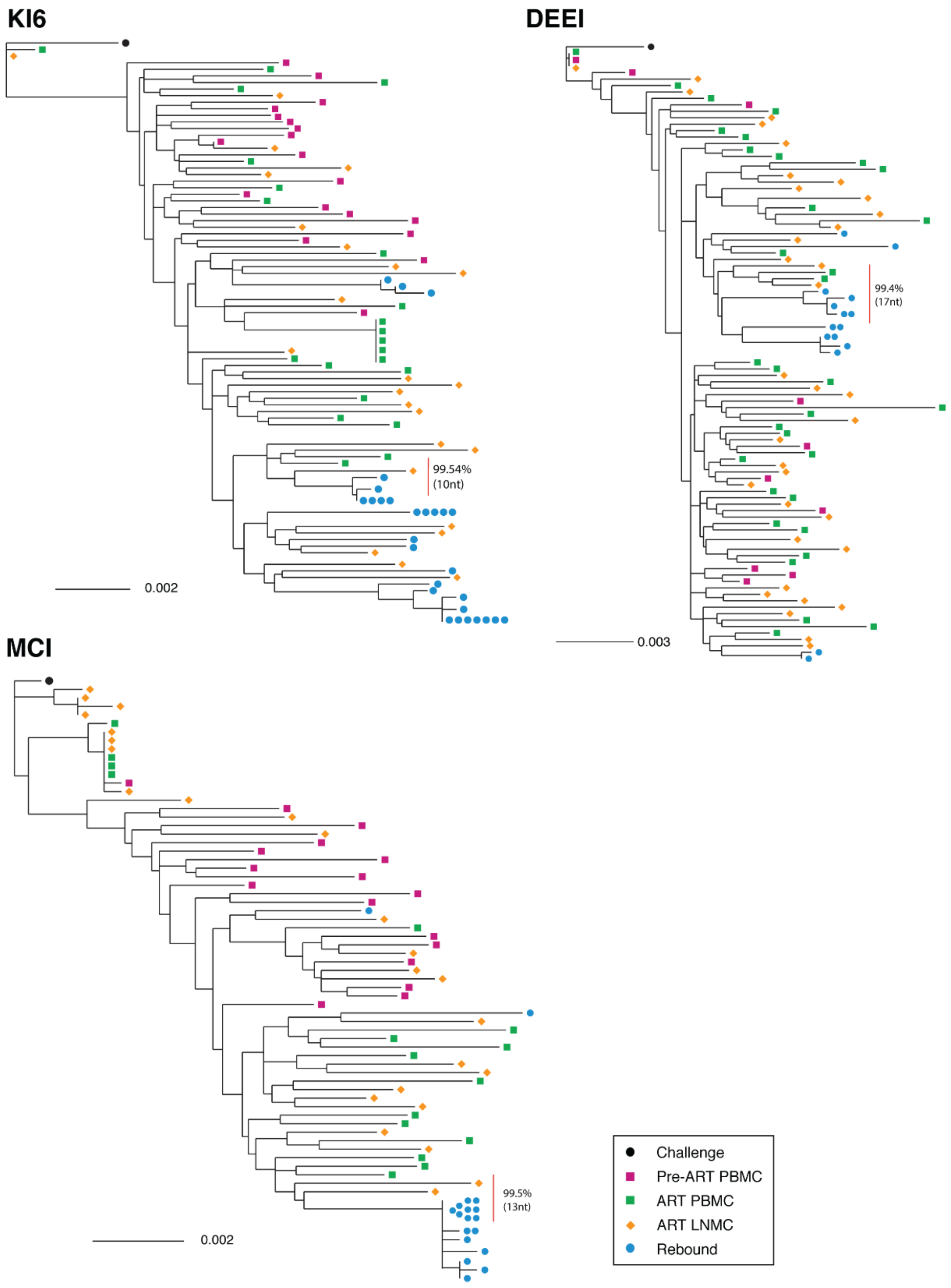

Supplementary Figure 7. Initial rebound viral RNA sequences compared with rcNFL viral DNA sequences from Pre-ART PBMC, ART PBMC and ART LNMC. Phylogenetic trees of initial plasma rebound viral RNA sequences following ART discontinuation and rcNFL viral DNA sequences from Pre-ART PBMC, ART PBMC and ART LNMC from all 16 animals. The closest matches are indicated with a vertical red line with the sequence identity indicated.

Parental/Input	rcNFL in PBMC				
Recombination	Initial Rebound				
	Total # of Recombinants	Duplicates	Unique # of Recombinants	Total # of Initial Rebound	Percentage
1. KPN	0	0	0	17	0%
2. GA43	0	0	0	22	0%
3. DEAE	0	0	0	15	0%
4. KI6	0	0	0	28	0%
5. MCI	0	0	0	19	0%
6. DEEI	0	0	0	15	0%
7. DEL5	0	0	0	13	0%
8. DEG2	0	0	0	13	0%
9. PII	1	0	1	25	4%
10. PZB	0	0	0	24	0%
11. PZX	0	0	0	27	0%
12. DEAB	0	0	0	10	0%
13. DEKW	3	0	3	15	20%
14. DEP0	2	0	2	10	20%
15. KIC	0	0	0	21	0%
16. GB97	0	0	0	29	0%

Parental/Input	rcNFL in LNMC				
Recombination	Initial Rebound				
	Total # of Recombinants	Duplicates	Unique # of Recombinants	Total # of Initial Rebound	Percentage
1. KPN	0	0	0	17	0%
2. GA43	0	0	0	22	0%
3. DEAE	0	0	0	15	0%
4. KI6	0	0	0	28	0%
5. MCI	0	0	0	19	0%
6. DEEI	0	0	0	15	0%
7. DEL5	0	0	0	13	0%
8. DEG2	0	0	0	13	0%
9. PII	0	0	0	25	0%
10. PZB	0	0	0	24	0%
11. PZX	0	0	0	27	0%
12. DEAB	0	0	0	10	0%
13. DEKW	0	0	0	15	0%
14. DEP0	2	0	2	10	20%
15. KIC	0	0	0	21	0%
16. GB97	0	0	0	29	0%

Parental/Input	Initial Rebound				
Recombination	Post-peak virus				
	Total # of Recombinants	Duplicates	Unique # of Recombinants	Total # of Post-peak virus	Percentage
1. KPN	0	0	0	0	0.0%
2. GA43	5	1	4	15	26.7%
3. DEAE	0	0	0	0	0.0%
4. KI6	7	0	7	26	26.9%
5. MCI	4	3	1	23	4.3%
6. DEEI	5	1	4	10	40.0%
7. DEL5	0			13	0.0%
8. DEG2	4	3	1	8	12.5%
9. PII	25	6	19	33	57.6%
10. PZB	16	0	16	27	59.3%
11. PZX	9	6	3	28	10.7%
12. DEAB	9	6	3	16	18.8%
13. DEKW	17	1	16	18	88.9%
14. DEP0	6	2	4	18	22.2%
15. KIC	11	5	6	33	18.2%
16. GB97	6	2	4	37	10.8%

Supplementary Figure 8. Percentage of recombinants events. The percentage of recombination events was calculated by dividing the number of unique recombinants of total initial rebound virus or post-peak rebound virus.

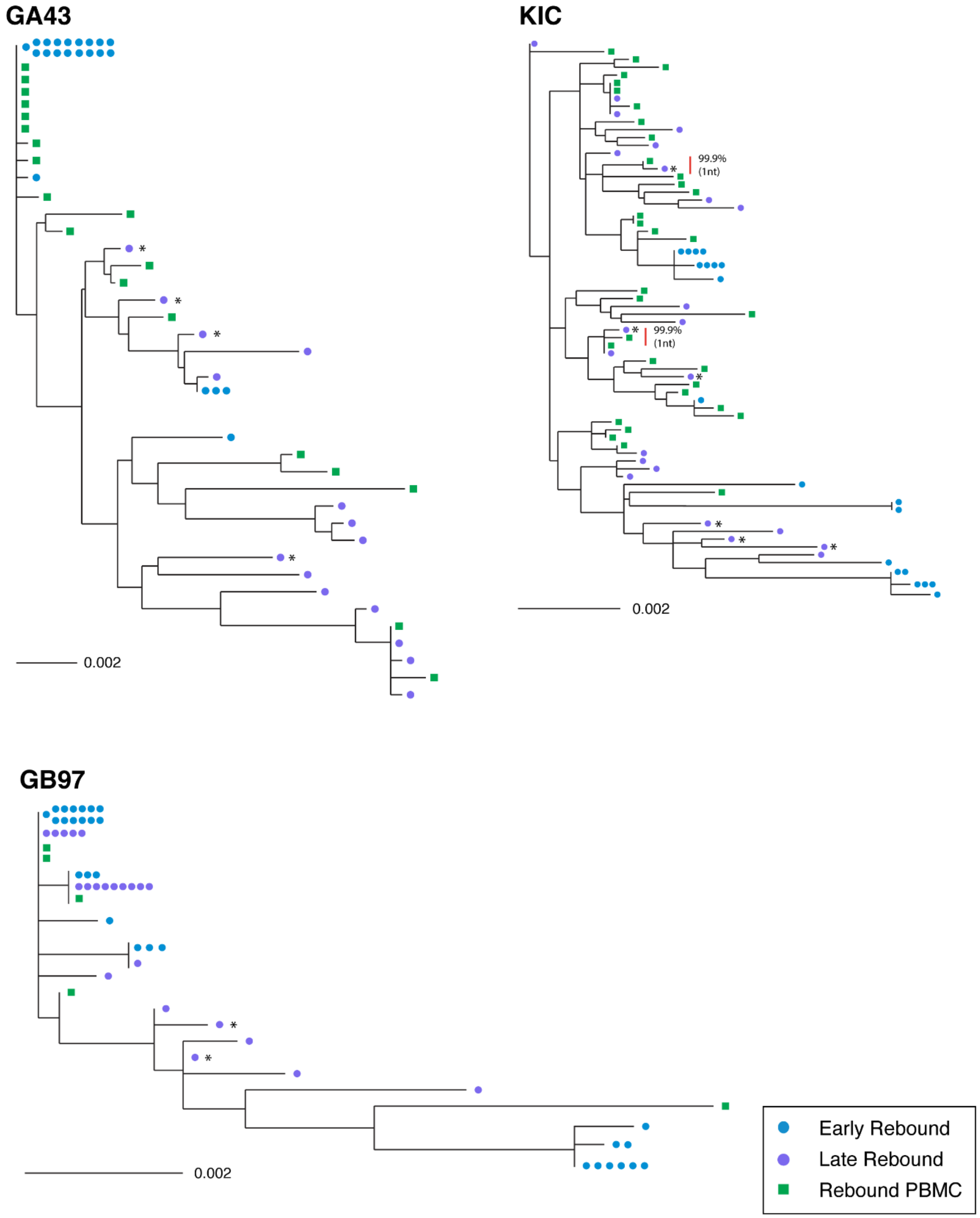

Supplementary Figure 9. Post-peak rcNFL viral DNA in PBMC compared with initial rebound and post-peak viral RNA sequences. Phylogenetic trees of post-peak rcNFL viral DNA in PBMC and viral RNA of initial rebound and post-peak virus. Post-peak recombinants were labeled with an asterisk. The closest matches are indicated with a vertical red line with the sequence identity indicated.
