# Supplementary material for: Origin of rebound virus in chronically SIV-infected Rhesus monkeys following treatment discontinuation
Source: Nat Commun. 2020 Oct 27;11:5412. doi: 10.1038/s41467-020-19254-2 (PMC7591481; doi:10.1038/s41467-020-19254-2)
Supplement: Supplementary file 2 — Reporting Summary [file 41467_2020_19254_MOESM2_ESM.pdf]

nature research

Corresponding author(s): Dan Barouch

Last updated by author(s): Sep 25, 2020

Reporting Summary

Nature Research wishes to improve the reproducibility of the work that we publish. This form provides structure for consistency and transparency in reporting. For further information on Nature Research policies, see Authors & Referees and the Editorial Policy Checklist .

Statistics

For all statistical analyses, confirm that the following items are present in the figure legend, table legend, main text, or Methods section.

n/a	Confirmed	
☐	☒	The exact sample size (*n*) for each experimental group/condition, given as a discrete number and unit of measurement
☐	☒	A statement on whether measurements were taken from distinct samples or whether the same sample was measured repeatedly
☐	☒	The statistical test(s) used AND whether they are one- or two-sided<br>*Only common tests should be described solely by name; describe more complex techniques in the Methods section.*
☐	☒	A description of all covariates tested
☒	☐	A description of any assumptions or corrections, such as tests of normality and adjustment for multiple comparisons
☐	☒	A full description of the statistical parameters including central tendency (e.g. means) or other basic estimates (e.g. regression coefficient) AND variation (e.g. standard deviation) or associated estimates of uncertainty (e.g. confidence intervals)
☐	☒	For null hypothesis testing, the test statistic (e.g. *F*, *t*, *r*) with confidence intervals, effect sizes, degrees of freedom and *P* value noted<br>*Give P values as exact values whenever suitable.*
☒	☐	For Bayesian analysis, information on the choice of priors and Markov chain Monte Carlo settings
☒	☐	For hierarchical and complex designs, identification of the appropriate level for tests and full reporting of outcomes
☒	☐	Estimates of effect sizes (e.g. Cohen's *d*, Pearson's *r*), indicating how they were calculated

Our web collection on statistics for biologists contains articles on many of the points above.

Software and code

Policy information about availability of computer code

Data collection	No software was used
Data analysis	GraphPad Prism v8.3, MEGA-X 10.1, Sequencher 5.4.6, Geneious Prime 2020.1, ElimDupes (no version information), Hypermut (no version information), RAPR (no version information), Highlighter (no version information), GeneCutter (no version information).

For manuscripts utilizing custom algorithms or software that are central to the research but not yet described in published literature, software must be made available to editors/reviewers. We strongly encourage code deposition in a community repository (e.g. GitHub). See the Nature Research guidelines for submitting code & software for further information.

Data

Policy information about availability of data

All manuscripts must include a data availability statement. This statement should provide the following information, where applicable:

- Accession codes, unique identifiers, or web links for publicly available datasets
- A list of figures that have associated raw data
- A description of any restrictions on data availability

All data are available in the Article and its Supplementary Information. Source data with unique identifiers are provided with this paper. LANL Database tools were used for sequence analysis in this study (www.hiv.lanl.gov). All sequences have been deposited in GenBank with accession numbers MN884181-MN88543 and MW004671-MW004840.

Field-specific reporting

Please select the one below that is the best fit for your research. If you are not sure, read the appropriate sections before making your selection.

Life sciences study design

All studies must disclose on these points even when the disclosure is negative.

Sample size	No formal sample size calculation was performed. A sample size of N=4-7 per group was selected based on our experience to date to achieve power to detect a 2-fold increase in time to viral rebound (Borducchi et al, Nature, 2016 and Borducchi et al, Nature, 2018).
Data exclusions	No data were excluded.
Replication	Virologic and immunologic measures were performed in duplicate. Technical replicates were minimally different. Multiple time points were assayed to ensure reproducibility.
Randomization	Animals expressing protective MHC class I alleles were excluded and susceptible and resistant TRIM5 alleles were distributed among the groups. Animals were divided into groups based on baseline viral loads, and were otherwise randomly allocated to groups.
Blinding	Immunologic and virologic assays were performed blinded.

Reporting for specific materials, systems and methods

We require information from authors about some types of materials, experimental systems and methods used in many studies. Here, indicate whether each material, system or method listed is relevant to your study. If you are not sure if a list item applies to your research, read the appropriate section before selecting a response.

Materials & experimental systems

n/a	Involved in the study	
☒	☐	Antibodies
☒	☐	Eukaryotic cell lines
☒	☐	Palaeontology
☐	☒	Animals and other organisms
☒	☐	Human research participants
☒	☐	Clinical data

Methods

n/a	Involved in the study	
☒	☐	ChIP-seq
☒	☐	Flow cytometry
☒	☐	MRI-based neuroimaging

Animals and other organisms

Policy information about studies involving animals; ARRIVE guidelines recommended for reporting animal research

Laboratory animals	Outbred Indian-origin adult male and female rhesus macaques (Macaca mulatta), 6-12 years old
Wild animals	The study did not involve wild animals.
Field-collected samples	The study did not involve samples collected from the field.
Ethics oversight	All monkey studies were approved by the Alpha Genesis Institutional Animal Care and Use Committee (IACUC).

Note that full information on the approval of the study protocol must also be provided in the manuscript.
